# Stage-Specific Characterization of Physiological Response to Heat Stress in the Wheat Cultivar Norin 61

**DOI:** 10.3390/ijms22136942

**Published:** 2021-06-28

**Authors:** Sachiko Matsunaga, Yuji Yamasaki, Yusuke Toda, Ryosuke Mega, Kinya Akashi, Hisashi Tsujimoto

**Affiliations:** 1 United Graduate School of Agricultural Sciences, Tottori University, 4-101 Koyama-cho Minami, Tottori 680-8553, Japan; smatsunaga3005.research@gmail.com (S.M.); akashi.kinya@tottori-u.ac.jp (K.A.); 2 Arid Land Research Center, Tottori University, 1390 Hamasaka, Tottori 680-0001, Japan; yujyamas@tottori-u.ac.jp; 3 Department of Agricultural and Environmental Biology, Graduate School of Agricultural and Life Sciences, The University of Tokyo, 1-1-1 Yayoi, Bunkyo, Tokyo 113-8657, Japan; yusuke0320.research@gmail.com; 4 Graduate School of Science & Technology for Innovation, Yamaguchi University, 1677-1 Yoshida, Yamaguchi 753-8515, Japan; mega@yamaguchi-u.ac.jp

**Keywords:** heat priming, acclimatization, yield, node, seed maturation, photosynthesis, arid region

## Abstract

Bread wheat (*Triticum aestivum*) is less adaptable to high temperatures than other major cereals. Previous studies of the effects of high temperature on wheat focused on the reproductive stage. There are few reports on yield after high temperatures at other growth stages. Understanding growth-stage-specific responses to heat stress will contribute to the development of tolerant lines suited to high temperatures at various stages. We exposed wheat cultivar “Norin 61” to high temperature at three growth stages: seedling–tillering (GS1), tillering–flowering (GS2), and flowering–maturity (GS3). We compared each condition based on agronomical traits, seed maturity, and photosynthesis results. Heat at GS2 reduced plant height and number of grains, and heat at GS3 reduced the grain formation period and grain weight. However, heat at GS1 reduced senescence and prolonged grain formation, increasing grain weight without reducing yield. These data provide fundamental insights into the biochemical and molecular adaptations of bread wheat to high-temperature stresses and have implications for the development of wheat lines that can respond to high temperatures at various times of the year.

## 1. Introduction

The world’s cereal production must continue to increase under global warming to meet population increases. Rice, maize, and wheat account for 90% of the current total cereal production. Of these, wheat is most susceptible to heat stress [1]. Asseng et al. [2] reported a 6% reduction in yield for every 1 °C increase in temperature. From an analysis of temperatures and yields in Sudan, the hottest wheat-growing area in the world, Iizumi et al. [3] concluded that the development of heat-tolerant wheat cultivars is essential.

Plants show a variety of morphological, physiological, biochemical, and molecular changes when exposed to high temperatures. Typical responses include growth disorders, inhibition of photosynthesis, accumulation or reduction of plant hormones, production of heat shock proteins, and accumulation of reactive oxygen species [4]. Wheat shows such responses. Reduction of photosynthetic capacity caused by chlorophyll loss and sterility caused by abnormal development of pollen grains or tubes greatly reduce grain yield [5]. To overcome heat stress, wheat plants can lower canopy temperature by opening stomata to activate transpiration [6]. Winter wheat grows where the temperature rises with crop growth. Previous studies of the effect of high temperatures on wheat have focused on the most sensitive stage, i.e., the reproductive stage. However, in future global warming scenarios, temperatures will increase in all growth stages [7]. Heat-stress effects might depend on the growth stage [8]. Thus, it is necessary to consider responses to high temperatures during other growth stages as well. To breed cultivars that maintain yield even under warming conditions, it is necessary to analyze the effects of heat on grain yield. However, few studies have comprehensively evaluated the morphological and physiological responses of yield potential to high temperatures at the very hot temperatures prevalent in some wheat cultivation areas.

Here, we observed morphological and physiological responses of the Japanese bread wheat cultivar “Norin 61” exposed to high temperature at three growth stages: from seedling to tillering (GS1), from tillering to flowering (GS2), and from flowering to maturity (GS3). Our aim was to elucidate the effects of high-temperature stress on agronomic traits and physiological responses during seed formation. The complete genome sequence of “Norin 61” has been assembled in the 10+ Genome Decoding Project [9,10,11]. We used “Norin 61” because, in our previous studies in Wad Medani, Sudan, the hottest wheat-growing area in the world [12,13], we unexpectedly found that it was at least as tolerant to high temperatures as current Sudanese cultivars.

## 2. Results

### 2.1. Stage-Specific Response to Heat on Grain Yield

**Figure 1:**
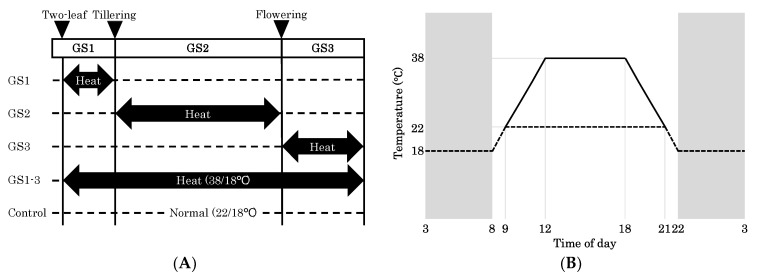
Outline of heat stress experiment. Wheat plants were kept at 22 °C day/18 °C night (Normal, Control) or exposed to transient high temperature (Heat) at 38/18 °C at each growth stage. (**A**) Arrows indicate the high-temperature treatment and dashed lines indicate normal growth conditions. (**B**) Daily temperature variation. White background, day; gray, night.

**Figure 2:**
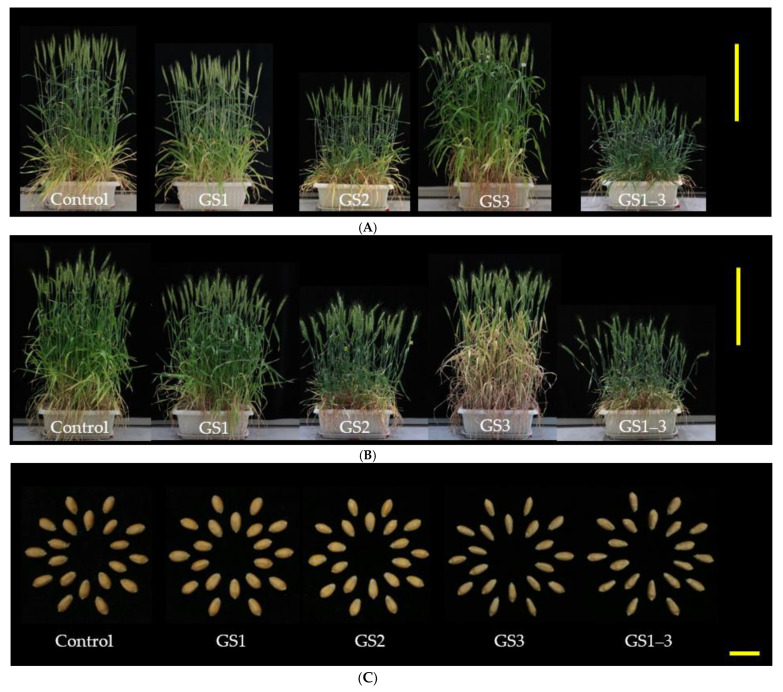
Effect of high temperature during each growth stage on plant and seed morphology. (**A**,**B**) Each plant at (**A**) anthesis and (**B**) 14 days post-anthesis. (**C**) Harvested seeds. Bars: plants, 50 cm; seeds, 1 cm.

**Figure 3:**
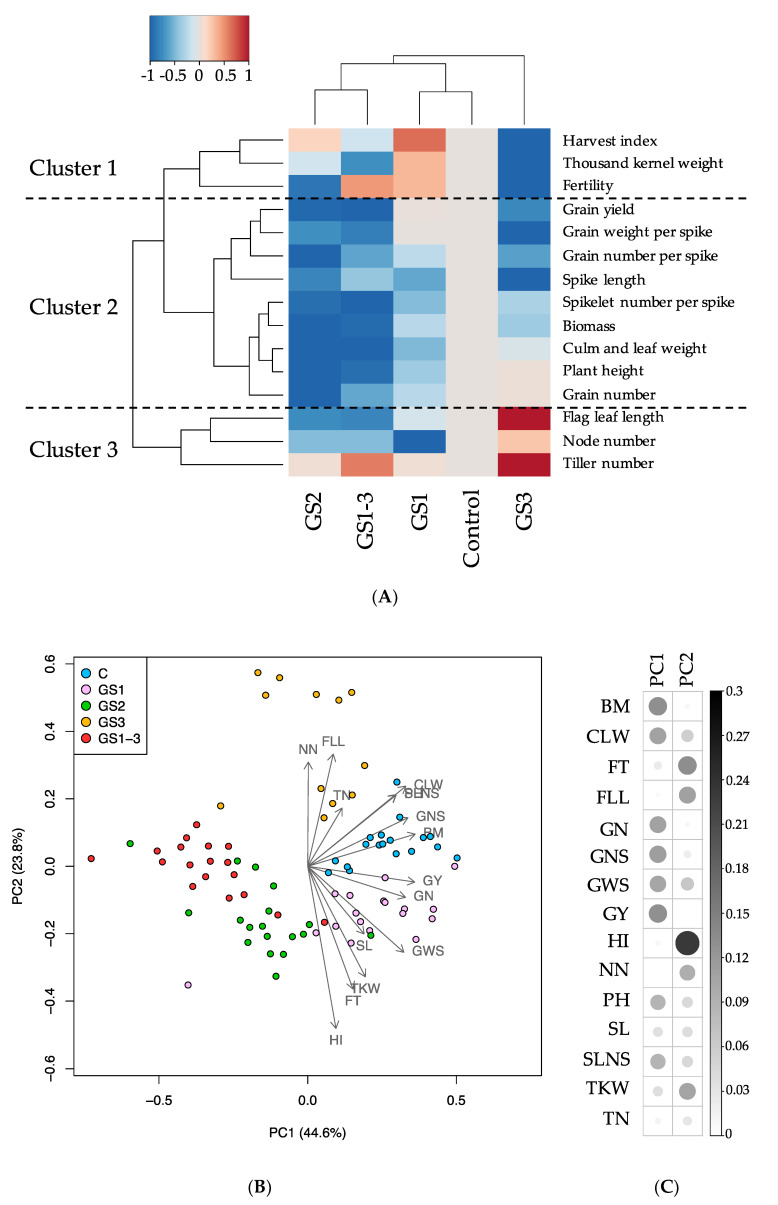
Hierarchical clustering and heat map (**A**), principal component analysis (PCA) (**B**) and trait contribution to PC1 and PC2 (**C**) of agricultural traits in each growing condition (GS1, GS2, GS3, GS1-3 and control indicated by C). Colors in (**A**) show increase (red) or decrease (blue) compared with the control. In (**C**), the strength of the contribution is indicated by the size and intensity of each circle. In (**B**,**C**), BM, Biomass; CLW, Culm and leaf weight; FT, Fertility; FLL, Flag leaf length; GN, Grain number; GNS, Grain number per spike; GWS, Grain weight per spike; GY, Grain yield; HI, Harvest index; NN, Node number; PH, Plant height; SL, Spike length; SLNS, Spikelet number per spike; TKW, Thousand kernel weight; TN, Tiller number.

After exposure of plants to high temperatures during GS1, GS2, GS3, or all stages (GS1–3) (Figure 1), we investigated agricultural traits. We found stage-specific alterations in plant and seed morphology (Figure 2); seed size of plants exposed to heat during ripening (GS3 and GS1–3) was clearly reduced, while that of plants exposed during GS1 was increased (Figure 2C). The traits formed three clusters (Figure 3A): (1) those positively affected by heat during GS1, (2) those negatively affected by heat during all stages, and (3) those affected negatively by heat during GS1 and positively during GS3.

**Figure 4:**
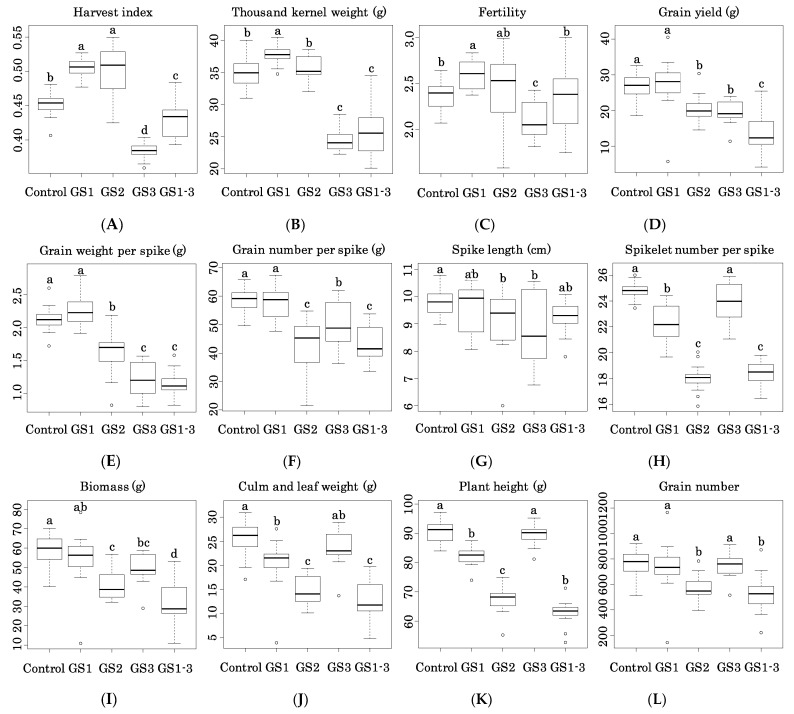

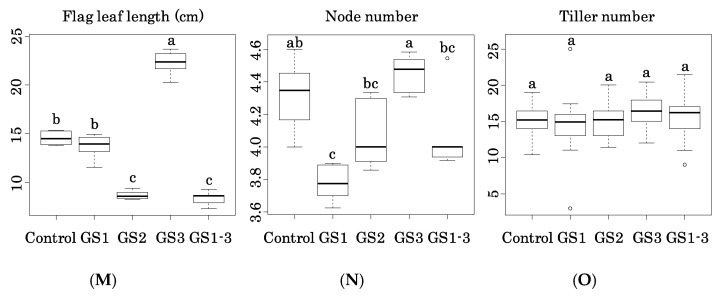
Differences in agricultural traits of heat-treated plants from the control. (**A**) Harvest index, (**B**) Thousand kernel weight (g), (**C**) Fertility, (**D**) Grain yield (g), (**E**) Grain weight per spike (g), (**F**) Grain number per spike, (**G**) Spike length (cm), (**H**) Spikelet number per spike, (**I**) Biomass (g), (**J**) Culm and leaf weight (g), (**K**) Plant height (cm), (**L**) Grain number, (**M**) Flag leaf length (cm), (**N**) Node number and (**O**) Tiller number. The box plots are based on corrected data from two seasons. Bars with the same letter are not significantly different by Tukey’s range test (*p* < 0.05). *n* = 12.

Heat during GS1 increased harvest index (HI, by 12% relative to control), thousand-kernel weight (TKW, by 7.5%), and fertility (FT, by 10%) (Figure 4A–C). Heat during GS2 increased HI (to the same level as in GS1), but did not affect TKW or FT. Heat during GS3, on the other hand, reduced these values (HI, −15%; TKW, −30%; FT, −11%). Heat during all three stages (GS1–3) decreased HI (by −5%) and TKW (by −27%), indicating that the decreases caused by heat during GS3 canceled the increases caused by heat during GS1.

Heat during GS1 had little or no effect on grain yield (GY), grain weight per spike (GWS), grain number per spike (GNS), or spike length (SL). Heat during GS2, GS3, and GS1–3, however, reduced these values (Figure 4D–G). Heat during GS2 and GS3 similarly reduced GY (GS2, by −24%; GS3, by −28%) and SL (GS2, by −6%; GS3, by −9%), but differentially reduced GWS (GS2, by −23%; GS3, by −43%) and GNS (GS2, by −26%; GS3, by −15%). Thus, heat affected these traits during GS1–3. All growth stages had cumulative effects on GY (−49%), but GS3 explained most of the effect on GWS (−46%), and GS2 explained most of the effect on GNS (−27%).

Heat during GS1, GS2, and GS1–3 reduced spikelet number per spike (SLNS), biomass (BM), culm and leaf weight (CLW), plant height (PH), and grain number (GN), but heat during GS3 did not (Figure 4H–L). The effect was greater during GS2 than during GS1. The effects were additive. Heat during GS2 reduced SLNS by 27%, BM by 31%, CLW by 43%, PH by 26%, and GN by 26%. Heat during GS1 also reduced SLNS, CLW, and PH. Heat during GS1 and GS2 reduced PH, while heat during GS1–3 had an effect on heat during GS2, as can be seen from plant morphology (Figure 2A). On the other hand, heat during GS2 and GS3 reduced BM, with a multiplier effect during GS1–3. Since CLM did not differ significantly between GS3 and the control, BM decrease during GS3 is attributable to GY.

Heat during GS3 increased flag leaf length (FLL) but not node number (NN) or tiller number (TN). Heat during GS1 and GS2 reduced NN, and heat during GS2 reduced FLL (Figure 4M,N) but had no significant effect on TN (Figure 4O).

Principal component analysis (PCA) showed that each condition was separated, but GS2 and GS1-3 were placed close to each other (Figure 3B). GS2 and other traits were separated along with agronomic traits such as BM, GY, and GNS in PC1, and GS3 was separated along with HI in PC2 (Figure 3C).

**Figure 5:**
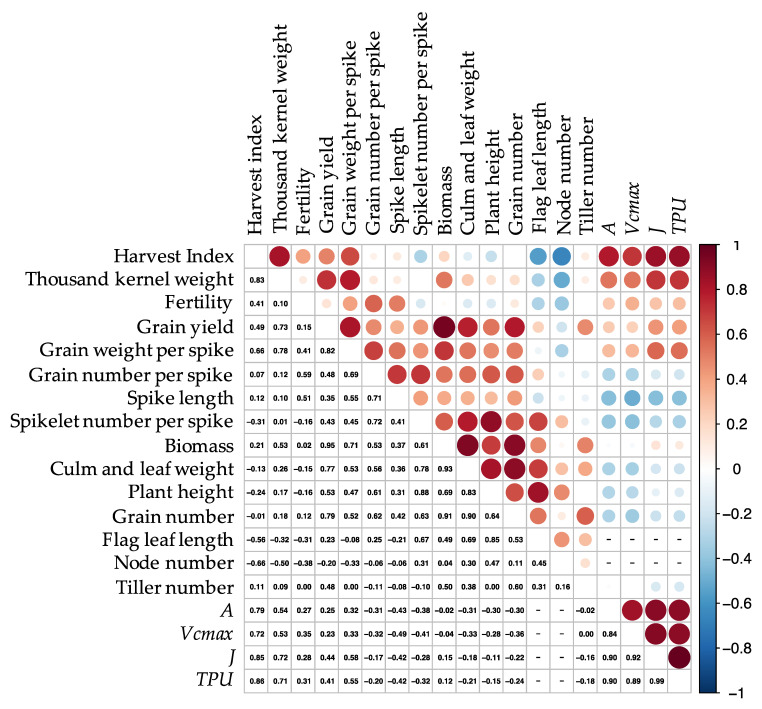
Heat map of correlation matrix of agricultural traits and physiological traits related to photosynthetic rate. Correlation coefficients were calculated without separating growing conditions. The strength of the correlation is indicated by the color, size, and intensity of each circle: red = positive, blue = negative; larger and darker = higher, smaller and lighter = lower. *A*, photosynthetic CO_2_ assimilation; *Vcmax*, the maximum carboxylation rate of rubisco; *J*, the rate of photosynthetic electron transport; *TPU*, the triose phosphate utilization. Bar (-) indicates that the corresponding data do not exist.

There were strong phenotypic correlations of GY with BM (*r* = 0.95) and GWS (0.82); of PH with CLW (0.83) and FLL (0.85); of BM with CLW (0.93) and GN (0.91); and of HI with TKW (0.83) (Figure 5). There were negative correlations of HI with FLL (−0.56), and of FT with TN (−0.51). It is noteworthy that NN and HI were negatively correlated (−0.66), though these two traits seem not to be directly related. Physiological traits related to photosynthetic rate are also included in Figure 5 (See in Section 2.3).

### 2.2. Effect of Heat on Seed Development

**Figure 6:**
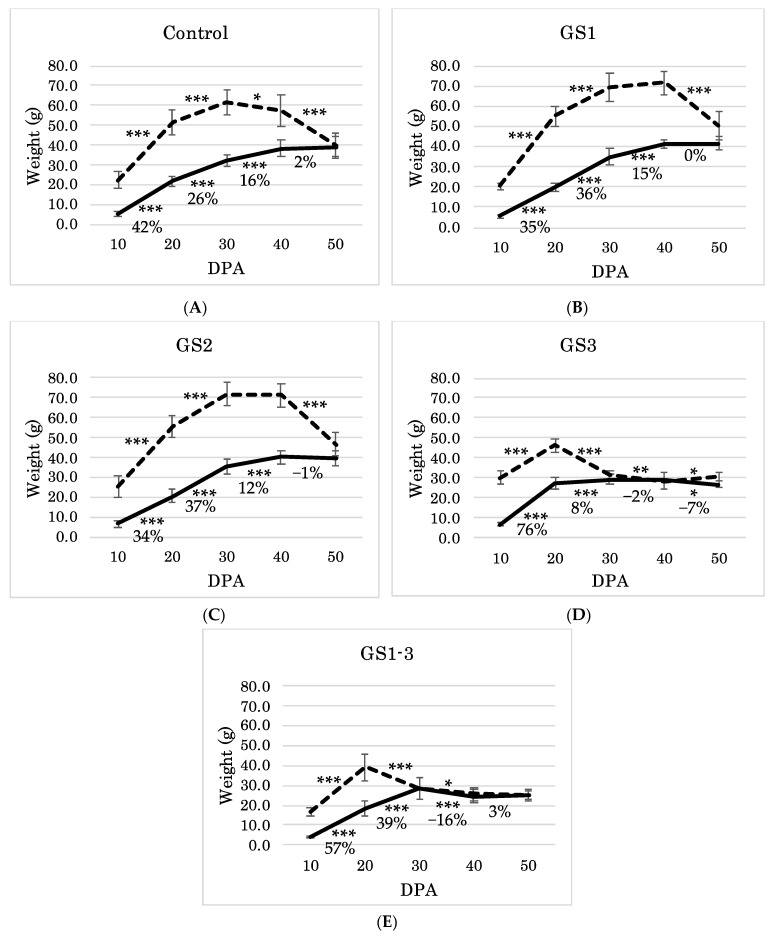
Time-series transition of seed maturity every 10 days from flowering date based on fresh weight (- - -) and dry weight (—). * Significant differences between pairs of values (* *p* = 0.05; ** *p* = 0.01; *** *p* = 0.001). Bonferroni’s correction was applied to control *p*-values in multiple comparisons. The percentages indicate the proportion of dry weight at each point calculated with an average dry weight at 50 DPA of 100%. (**A**) Control and (**B**–**E**) samples treated at high temperature during (**B**) GS1, (**C**) GS2, (**D**) GS3, and (**E**) all growth stages (GS1–3). *n* = 3.

We measured both fresh and dry seed weight (FW, DW). FW of seeds on the control plants increased up to 30 days post-anthesis (DPA) and then decreased until 50 DPA (Figure 6A). The seeds gained 42% of the final DW between 10 and 20 DPA, and a further 16% between 30 and 40 DPA despite desiccation. This indicates that the translocation of anabolic products continued into this period. FW equaled DW by 50 DPA with the completion of desiccation.

FW of seeds on plants exposed to heat during GS1 showed a similar trend to the control (Figure 6B), but decreased between 40 and 50 DPA (Figure 6B). Heat during GS1 thus delayed the start of desiccation. This delay may cause the increase of HI, TKW, and FT in those plants (Figure 4). As in the control, FW of seeds on plants exposed to heat during GS2 increased till 30 DPA and began decreasing from 40 DPA (Figure 6C). Filling of seeds continued even between 30 and 40 DPA.

In contrast, seeds of plants exposed to heat during the seed formation stage (GS3 and GS1–3) had the highest FW and DW at 20 DPA. In this short period (10–20 DPA), the seeds gained as much as 76% and 57%, respectively, of the final DW at 50 DPA. DW increased till 30 DPA, but FW decreased from 20 DPA (Figure 6D,E). We attribute the decrease of HI, TKW, and FT to this short period.

### 2.3. Heat Treatment at Seedling Stage Increases Carbon Assimilation Period

**Figure 7:**
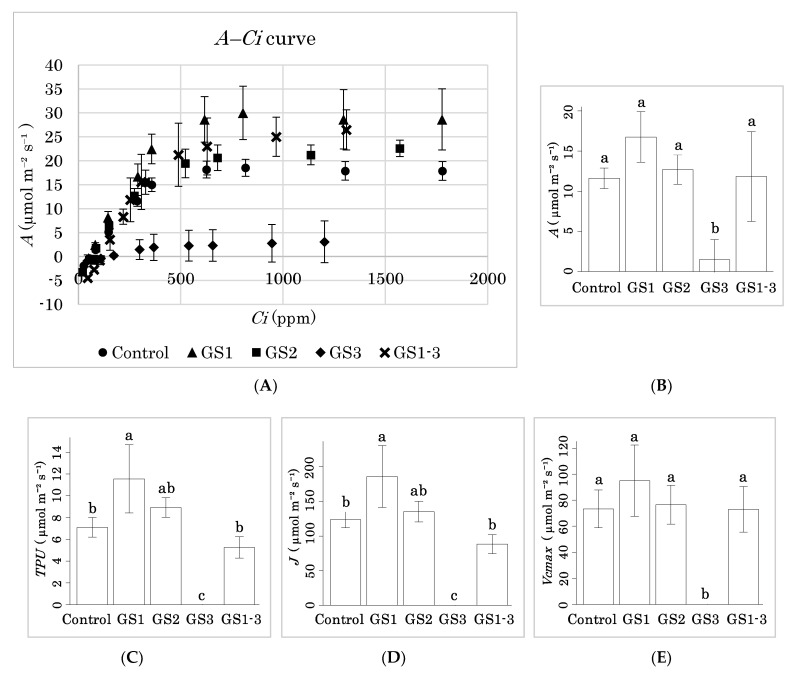

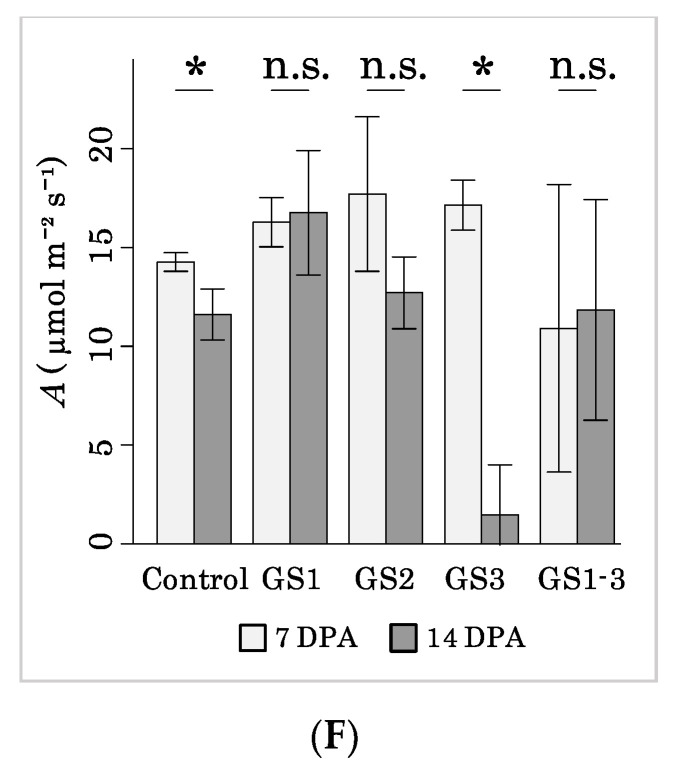
Photosynthetic rate measured at 14 DPA. (**A**) *A–C_i_* curve; (**B**) carbon assimilation rate, (**C**) *TPU*, (**D**) *J*, and (**E**) *Vcmax* at 400 ppm CO_2_; (**F**) *A* at 400 ppm CO_2_ at 7 and 14 DPA (* *p* = 0.01). *n* = 3. Bars with the same letters in B–F are not significantly different by Tukey’s range test (*p* < 0.05).

**Figure 8:**
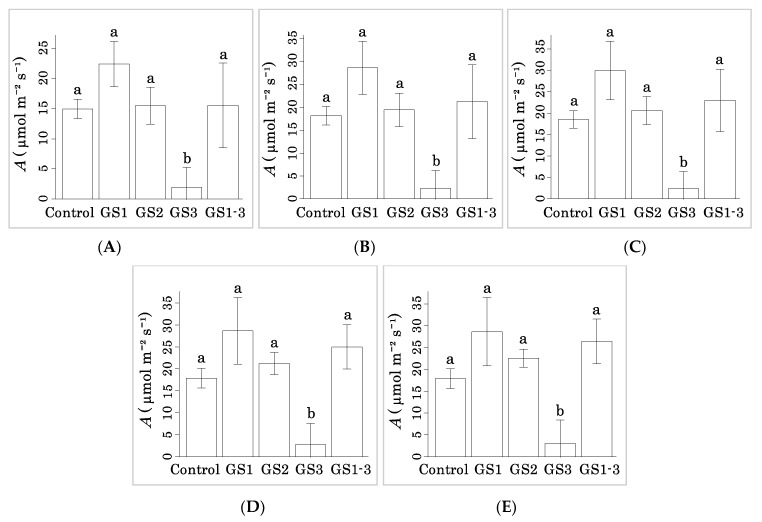
Carbon assimilation rate at CO_2_ concentrations of (**A**) 500 ppm, (**B**) 800 ppm, (**C**) 1000 ppm, (**D**) 1500 ppm, and (**E**) 2000 ppm. Letters on the bars with the same alphabet indicate not significant difference by Tukey’s range test (*p* < 0.05).

The above results show that heat exposure during GS1 prolongs the seed maturation period. To understand how, we measured photosynthetic CO_2_ assimilation (*A*) at 14 DPA in plants exposed at different stages (Figure 7A). Plants exposed to heat during GS1 tended to have a higher *A* than the control at 400 ppm CO_2_ (Figure 7B). Plants exposed during GS2 had a similar *A* to the control. However, plants exposed during GS3 had extremely low *A*: two of the three replicates reached complete leaf senescence (Figure 2B) and the other had extremely low *A* at 14 DPA. Interestingly, plants exposed to heat during GS1–3 had similar *A* to the control (Figure 7A) despite the measurement of photosynthesis in the hot chamber during GS3. This trend was apparent under high CO_2_ concentrations, and *A* of plants exposed to heat during GS1 remained higher than the control at all concentrations (Figure 8).

We calculated the triose phosphate utilization (*TPU*), the rate of photosynthetic electron transport (*J*), and the maximum carboxylation rate of rubisco (*Vcmax*) at 400 ppm [14] from the photosynthetic values (Figure 7C–E). *TPU* and *J* were significantly higher than the control in the GS1 sample. All three were zero in the GS3 sample. *A* was significantly lower in the control and GS3 at 14 DPA than at 7 DPA, and GS2 also showed a decreasing trend (Figure 7F). However, GS1 and GS1–3 maintained their photosynthetic activity.

This indicates that heat during GS1 and GS2 acclimatizes the plants to heat in the early seed maturation stage.

There were strong positive correlations in each physiological trait related to photosynthetic rate (*r* > 0.8). Additionally, there were strong correlations of HI with *A* (*r* = 0.79) and *Vcmax* (0.72) and *J* (0.85) and *TPU* (0.86); of TKW with *J* (0.72) and *TPU* (0.71) (Figure 5).

## 3. Discussion

### 3.1. Agronomic Traits Related to GY

GY was reduced by reduction of BM, PH, and GN due to heat during GS2 and by reduction of GWS, GNS, FT, HI, and TKW due to heat during GS3 (Figure 2 and Figure 3). The similar losses caused by heat during GS2 and GS3 were additive; therefore, heat during GS1–3 had a more severe effect. Most traits were positively correlated with each other such as PH-SLNS, but some were negatively correlated, such as HI–NN (Figure 5).

“Norin 61” has the photoperiod-insensitive *Ppd-D1a* allele and the semi-dwarfing *Rht-D1b* allele [11]. *Rht-D1b* is associated with high-temperature tolerance in Europe [15]. Gibberellic acid (GA)-sensitive (*Rht8*, *Rht12*, *rht*), semi-dwarf (*Rht-D1b*) plants at a high temperature below 36 °C at booting and anthesis stages maintained grain number per spikelet, while severely dwarf (*Rht-D1c*) plants had fewer grains [16]. However, heat stress above 36 °C eroded tolerance regardless of *Rht* allele. Our heat stress condition (38 °C) may erode the regulatory system for GA sensitivity of plant height and spikelet number regardless of *Rht* allele. Genetic analyses of near-isogenic wheat lines differing in plant height on account of *Ppd1* and *Rht* alleles showed pleiotropic effects on SLNS and PH [17,18]. The higher correlation between PH and SLNS obtained here suggests that the same mechanism regulates both traits (Figure 5).

Vegetative growth was strongly suppressed, leading to a reduction of BM in plants heat stressed during GS2. Decrease in vegetative BM limits source translocation to sink tissue during seed development. However, heat stress during GS2 down-regulated the volume of sink tissue by decreasing SLNS. As a result, TKW remained at the control level, and HI was improved.

Heat treatment before anthesis reduced GN [19,20,21]. At the double-ridge stage (the beginning of GS2), primordial spikelets begin to form in each spike [22]. A longer double-ridge phase increases SLNS, but increasing temperature above 19 °C decreases it [23,24]. GS1 does not span this period, which may be why GN was not reduced.

Heat treatment of 101 wheat cultivars at the booting stage decreased PH by about 10%, but that at heading and later had a much smaller effect, and a great reduction of TKW by heat stress after heading might decrease HI [25]. Other studies reported the reduction of HI, GY, and FT by heat stress during anthesis and grain development in wheat and rice [26,27]. We saw similar behavior here: heat during GS2 (from double ridge to heading) reduced PH, and heat during GS3 significantly decreased HI, TKW, FT, and GY (Figure 4A–D,K).

NN had a high negative correlation with HI. Nodes in grasses play the role of hubs in distributing nutrients taken up by the roots to various organs [28], and thus control the efficiency of translocation to grains. The reduction of NN might have eased the control of translocation to the grain. To confirm the control of translocation by nodes, we need to observe the distribution of element accumulation in plants.

#### 3.1.1. Duration of Grain Development Affected by Different Heat Stress Periods

The grains in the control treatment started desiccating at a mean of 609.9 °C·d (cumulative average daily temperature) (30 DPA) and were completely desiccated at 1016.5 °C·d (50 DPA). Those in the GS3 and GS1–3 treatments started desiccating at 593.4 °C·d (20 DPA) and were completely desiccated at 890.1 °C·d (30 DPA) [29]. The difference suggests that the start of desiccation depends on the accumulated average temperature during seed development. On the other hand, GS1 and GS2 extended the desiccation phase, even though they had the same temperature during seed development as the control (Figure 6). We consider that the timing of seed desiccation in the GS1 and GS2 treatments is regulated by mechanisms other than the cumulative average daily temperature. Previous research shows that the difference in the starch accumulation period between two wheat cultivars was regulated by a difference in abscisic acid (ABA) sensitivity at the start of desiccation [30]. In addition, epigenetic changes due to histone methylation and acetylation and expression changes due to retrotransposon insertion can alter the regulation of heat priming effects of heat [31,32,33]. For example, a heat-activated retrotransposon, *ONSEN* (*ATCOPIA78*), in Arabidopsis, generated a mutation of an ABA-responsive gene, leading to ABA insensitivity [34]. These studies suggest that heat stress treatment during GS1 and GS2 can remodel phytohormone sensitivity, thus changing seed development.

#### 3.1.2. Heat Treatment during GS1 Primed Later Grain Development

Heat stress during GS1 increased grain development duration and improved TKW and HI (Figure 4A,B and Figure 6). Studies of stress-related priming showed a short-term improvement by osmo-hardening, selenium, or salicylic acid treatment of germination and emergence in rice and a long-term improvement of yield and grain development [35,36,37]. Mendanha et al. [37] showed that 48 h of heat priming at the 3- or 5-leaf stage improved GY and TKW. Iizumi et al. [3] predicted that the optimal wheat sowing date under future global warming in Sudan is about 6 days earlier than at present. Our results show that even if earlier sowing before winter increases the risk of exposure to high temperatures, heat stress at the seedling stage does not significantly affect yield or yield-related traits, but rather improves seed formation.

#### 3.1.3. Improved Senescence of GS1 Heat-Primed Plants

As well as grain-filling in GS3 heat-treated plants, leaf senescence was accelerated, resulting in significantly reduced carbon assimilation, *J*, and *TPU* during 7 to 14 DPA (Figure 7B–E). This phenomenon is well characterized by loss of chlorophyll, low PSII activity, and reduced electron transport [38]. Control plants also reduced assimilation between 7 and 14 DPA, which accelerated leaf senescence. On the other hand, *A* was not significantly different between Control and GS1, but *TPU* and *J* had the highest values at 14 DPA in GS1 heat-treated plants. Time-series differences also suggest that senescence was delayed in GS1 heat-treated plants (Figure 6F). Tobacco lines with delayed senescence maintained significantly higher *J* and *TPU* and maintained rubisco activity [39]. This suggests that heat priming during GS1 suppresses the decrease in the final carbon assimilation rate by maintaining rubisco activity even at 14 DPA.

GS1–3 plants maintained higher photosynthetic activity than GS3 plants, maybe owing to acclimatization to a high temperature by exposure before the ripening stage. *A* decreased less in heat-primed plants than in unprimed plants [40], indicating that primed plants retain higher photosynthetic capacity under stress.

These behaviors may have led to the delayed senescence and prolonged starch deposition of source tissues during grain formation of GS1 plants, and ultimately contributed to the improvement of TKW.

## 4. Materials and Methods

### 4.1. Plant Material and Growth Conditions

The bread wheat (*Triticum aestivum* L.) cultivar “Norin 61” is a facultative wheat grown in Japan and is generally used as the standard check-in breeding programs there. It is the genetic background of the “MSD” population that we extensively used for a series of studies of heat tolerance in Sudan [12,41,42]. The fully matured seeds were harvested in 2016 in the field of the Agricultural Research Corporation, Wad Medani, Sudan, and had a >99% germination rate.

Plants were grown in climate chambers in which temperature, humidity, and light were controlled as below (Espec, Japan: W 1800 mm × D 1800 mm × H 2500 mm) at the Arid Land Research Center, Tottori University, Japan. Owing to limited space in the chambers, plants were grown in two seasons (March–July 2019 and January–May 2020). In the first experiment, one planter (46.5 cm × 23.7 cm × 17.5 cm) with six plants was used for each heat treatment. In the second experiment, two planters with six plants each were used. A control treatment was used in both seasons to standardize experimental fluctuations. Seeds were placed on a sheet of filter paper in Petri dishes and soaked in tap water. The seeds were stratified at 4 °C for 7 days and incubated for germination at room temperature for 72 h. Six 1-cm sprouts were transplanted into a planter filled with a commercial soil mixture made of composted bark, granular clay-like mineral, pumice, peat moss, perlite, and vermiculite (Cainz, Honjo City, Saitama, Japan). The planters were placed in the growth chambers. In the control treatment, the air temperature was controlled at 22 °C maximum in the daytime and 18 °C continuously at night. In the heat environments, it was controlled at 38 °C maximum during the day and 18 °C continuously at night (Figure 1A). The temperature shift slope was set at 4 °C per hour until these temperatures were reached. The light duration was 14 h light and 10 h dark (Figure 1B). The relative humidity was 30% day/50% night. The photosynthetic photon flux density was 1000 μmol s^−1^ m^−2^. Three growth stages were designated: GS1, heat exposure from two-leaf to tillering (Zadoks’s scale, Z12–Z19); GS2, heat exposure from budding to flowering (Z20–Z61), and GS3, from flowering to full maturity (Z62–Z95) (Figure 2A) [43,44]. Plants were transferred from the control chamber to the heating chamber during the respective periods. At the beginning of GS3, we supplied 10 g of NPK fertilizer (MC Ferticom Co., Ltd., Chiyoda Ward, Tokyo, Japan) containing 13% N, 16% P, and 16% K by total weight (present as water-soluble ammonium phosphate dibasic and water-soluble potassium).

### 4.2. Measurement of Plant Traits

Plant height from the soil surface to the top of the spike, excluding awns, was measured as PH. The number of tillers with spikes was counted as TN. The dry weight of the entire aboveground part was defined as BM, and that of the aboveground part without spikes was defined as CLW. Total grain yield from one plant was defined as GY. GWS, GNS, and SLNS were measured using five representative spikelets. HI was calculated as GY ÷ BM. FT was defined as GNS ÷ SLNS.

All six plants were measured independently in two experiments in the same condition. Six plants in the same planter of the first and second experiments were measured separately. After standardizing the values based on the average in the deferent experiments, the values from the total 12 plants as the replications. PCA, heat map and barplot of agronomical traits were created using R version 4.0.3 (https://www.R-project.org/, accessed on 5 April 2021).

To observe seed maturation, we excised seeds at 10, 20, 30, 40, and 50 DPA from the first and second florets of the ten spikelets above four or six spikelets, which may contain immature seeds, from the spike basal end. We measured FW immediately after collecting immature seeds and DW after complete desiccation in an oven at 80 °C. One spike per plant was collected from three of the six plants by random selection on each date. FW and DW are presented as the average of the three spikes.

### 4.3. Gas Exchange Rate Measurements

We measured the CO_2_ exchange rate of one flag leaf per plant selected from three of the six plants with an LI-6800XT portable gas exchange system (Li-Cor, Lincoln, NE, USA) at CO_2_ concentrations of 0, 50, 100, 200, 400, 500, 800, 1000, 1500, and 2000 ppm. The leaf temperature was 22 °C in the control and 38 °C in the heat treatment. Other settings were fixed: humidity, 18%; light intensity, 1000 μmol s^−1^ m^−2^ with 90% red and 10% blue light source; and flow rate, 500 μmol s^−1^. Three biological replicates in each environment were measured at 14 DPA. From the measurements, we plotted photosynthetic CO_2_ assimilation (*A*) and leaf intercellular CO_2_ concentration (*Ci*) (*A*–*Ci* curve). Through curve fitting, we obtained *Vcmax*, *J*, and *TPU* [14]. Data could not be collected from two GS3 replicates, which had completely been senesced.

## 5. Conclusions

In this study, we found the stage-specific heat response in the Japanese wheat cultivar “Norin 61”. Since the complete genome sequence of “Norin 61” was assembled, various information is available in this wheat cultivar. To conduct advanced research in this genetic background, many unique materials such as nested association mapping (NAM) populations and mutant panels were developed. Additionally, we generated a diverse wheat panel by incorporating the D genome from various *Aegilops tauschii* accessions into this variety and identified unique genomic markers associated with important agronomical traits. The findings in this study might give significant insights into the development of heat-tolerant wheat cultivars.

## Data Availability

Not applicable.
